# Sex Differences in the Behavioral and Synaptic Consequences of a Single *in vivo* Exposure to the Synthetic Cannabimimetic WIN55,212-2 at Puberty and Adulthood

**DOI:** 10.3389/fnbeh.2019.00023

**Published:** 2019-03-05

**Authors:** Milene Borsoi, Antonia Manduca, Anissa Bara, Olivier Lassalle, Anne-Laure Pelissier-Alicot, Olivier J. Manzoni

**Affiliations:** ^1^Aix Marseille Université, Institut National de la Santé et de la Recherche Médicale (INSERM), Institut de Neurobiologie de la Méditerranée (INMED), Marseille, France; ^2^Cannalab, Cannabinoids Neuroscience Research International Associated Laboratory, INSERM-Indiana University, Marseille, France; ^3^Assistance Publique Hôpitaux de Marseille (APHM), CHU Conception, Service de Psychiatrie, Marseille, France; ^4^Assistance Publique Hôpitaux de Marseille (APHM), CHU Timone Adultes, Service de Médecine Légale, Marseille, France

**Keywords:** prefrontal cortex, adolescence, cannabis, sexual differences, social behavior, CB1 receptor, synaptic plasticity, endocannabinoid

## Abstract

Heavy cannabis consumption among adolescents is associated with significant and lasting neurobiological, psychological and health consequences that depend on the age of first use. Chronic exposure to cannabinoid agonists during the perinatal period or adolescence alters social behavior and prefrontal cortex (PFC) activity in adult rats. However, sex differences on social behavior as well as PFC synaptic plasticity after acute cannabinoid activation remain poorly explored. Here, we determined that the consequences of a single *in vivo* exposure to the synthetic cannabimimetic WIN55,212-2 differently affected PFC neuronal and synaptic functions after 24 h in male and female rats during the pubertal and adulthood periods. During puberty, single cannabinoid exposure (SCE) reduced play behavior in females but not males. In contrast, the same treatment impaired sociability in both sexes at adulthood. General exploration and memory recognition remained normal at both ages and both sexes. At the synaptic level, SCE ablated endocannabinoid-mediated synaptic plasticity in the PFC of females of both ages and heightened excitability of PFC pyramidal neurons at adulthood, while males were spared. In contrast, cannabinoid exposure was associated with impaired long-term potentiation (LTP) specifically in adult males. Together, these data indicate behavioral and synaptic sex differences in response to a single *in vivo* exposure to cannabinoid at puberty and adulthood.

## Introduction

Cannabis is the most frequently and widely used illicit drug among adolescents in developed countries (Gowing et al., 2015). Heavy cannabis consumption among adolescents is associated with significant and lasting neurobiological, psychological and health consequences developing in a dose-dependent fashion which are influenced by age of first use (Iede et al., 2017; Levine et al., 2017; Lisdahl et al., 2018). Chronic adolescent exposure to cannabinoids is linked to persistent adverse effects such as poor cognitive and psychiatric outcomes in adulthood (Levine et al., 2017) and regular cannabis use is associated with psychosocial impairment even in users without cannabis use disorder (Foster et al., 2017).

The primary psychoactive compound of the plant *Cannabis sativa*, Δ-9-tetrahydrocannabinol (THC), as well as the main endogenous cannabinoids (eCBs) anandamide and 2-arachidonoylglycerol, all engage the same primary target in the central nervous system: the G-protein coupled cannabinoid receptor type 1 (CB1R). The eCB system consists of CB1R and other eCB receptors (e.g., CB2R, TRPV1R), eCB, and the enzymatic machinery for eCB synthesis and degradation (Hu and Mackie, 2015). It participates in neuronal development and synaptic plasticity in most brain areas (Gaffuri et al., 2012; Manduca et al., 2012; Lu and Mackie, 2016).

Social relationships during adolescence are essential for the maturation of adult social and cognitive skills (Casey et al., 2008). Disruptions in social exchanges participate to the etiology of neuropsychiatric and neurodevelopmental disorders (Hankin et al., 1998). In rodents, the eCB system modulates specific brain circuits underlying social behavior (Manduca et al., 2015, 2016; Wei et al., 2017) and rather unsurprisingly, exposure to cannabinoid agonists during adolescence alters social behavior at short- (Trezza and Vanderschuren, 2008a, 2009) and long-term time points (Rubino et al., 2008; Schneider, 2008; Schneider et al., 2008; Renard et al., 2016b). Current evidences suggest that “among the vast and complex neural networks involved in social behavior, the prefrontal cortex (PFC) and its massive reciprocal connections constitute a top-down modulatory system for social behavior” (Ko, 2017).

Sex differences in the eCB system (Cooper and Craft, 2018) appear early (Krebs-Kraft et al., 2010) and hormonal regulation affects eCB activity at adulthood: brain CB1R expression (Rodríguez de Fonseca et al., 1994; González et al., 2000) and eCB content (Bradshaw et al., 2006) are cycle-dependent in female rodents. Human studies demonstrate sex differences in cannabis use, acute and long-term effects, dependence and withdrawal. Males are more likely to initiate cannabis use at a younger age than female, and men use higher quantities more frequently than women who more frequently report nausea and anxiety during the withdrawal period (Stinson et al., 2006; Cuttler et al., 2016). During adolescence, women present a faster transition from initiation of cannabis use to regular use than men (Schepis et al., 2011). In rodents, the effects of cannabis also differ between sexes. Female rodents are more sensitive to the biphasic effects of cannabinoids on locomotion, have more impairments in spatial learning and are more sensitive to the reinforcing effects of cannabinoids than males (for review see Craft et al., 2013).

Many sex differences appear at adolescence during the maturation of adult social and cognitive skills (Casey et al., 2008; Rubino et al., 2008; Marusich et al., 2015; Rubino and Parolaro, 2015; Silva et al., 2016; Wiley et al., 2017). Adolescence is a period of profound morphological, neurodevelopmental and behavioral maturation. Brain volumes, sex steroids, and cortical morphometry all contribute to sex influences on developmental trajectories which are accompanied by changes in the behavioral repertoire normally observed in this transitional period from infancy to adulthood. Puberty is characterized by external physical signs and hormonal alterations whose onset is signaled by gonadotropin-releasing hormone (Spear, 2000; Harris and Levine, 2003; Ojeda et al., 2003). This period is elicited through the complex interaction of endogenous and environmental factors (Sisk and Foster, 2004). Both adolescence and puberty are essential periods of postnatal brain maturation and are characterized by heightened susceptibility to mental disorders (Schneider, 2013). Specifically, changes in puberty onset are associated with increased risk for depression, anxiety (Stice et al., 2001; Kaltiala-Heino et al., 2003) and substance use (Hummel et al., 2013).

Although the consequences of chronic exposure to cannabinoids during the adolescent period have been intensely studied (Liu et al., 2010; Cass et al., 2014; Lovelace et al., 2015; Rubino and Parolaro, 2015, 2016; Renard et al., 2016b), the neuronal and behavioral consequences of cannabis initiation, i.e., the first exposure to the drug, are less clear. Endocannabinoids are widespread mediators of synaptic plasticity, a phenomenon critical to normal function of neural circuits in several brain regions and experience-dependent adaptations (Castillo et al., 2012). As reviewed recently, cannabis has a brain-wide impact on synaptic functions and behavior including the PFC (Zlebnik and Cheer, 2016; Araque et al., 2017). We and others have previously revealed how a single exposure to THC *in vivo* ablates eCB-mediated synaptic plasticity (i.e., short and long-term depression, LTD) in the accumbens and hippocampus (Mato et al., 2004) but not hippocampal CA1 long-term potentiation (LTP; Hoffman et al., 2007) or eCB-LTD at VTA GABA synapses (Friend et al., 2017). Additionally, acute cannabinoid exposure impaired LTP in the ventral subiculum-accumbens pathway (Abush and Akirav, 2012). Thus, it appears that the effects of a single cannabinoid exposure (SCE) greatly depend on the brain area.

An important caveat is that most of the aforementioned studies used adolescent rats which range in age is between 25 and 45 days-old and do not take into account the pubertal period, i.e., its onset or completion. This interval is comprised of the different phases of adolescence which are common for males and females: early-, mid- and late-adolescence. However, mid-adolescence, when the physical markers of puberty typically appear, differs between sexes: females reach puberty around post-natal day (PND) 30–40 while puberty takes place in males later at approximately PND 40–50 (Schneider, 2008; Vetter-O’Hagen and Spear, 2012; Burke et al., 2017). Thus, based on the developmental profile of the eCB system and the sensitivity of the pubertal period, we reasoned that two factors, pubertal period and sex, may further complicate the effects of acute exposure to exogenous cannabinoids. The present study focuses on pubescent and adult rats of both sexes that were tested for social and cognitive behaviors as well as neuronal and synaptic parameters in pyramidal neurons of the PFC 24 h after a single *in vivo* exposure to the synthetic cannabimimetic WIN55,212-2.

## Materials and Methods

### Animals

Wistar rats bred in our animal facility were weaned from the mother at PND 21 and housed in groups of five individuals of the same sex with 12 h light/dark cycles and *ad libitum* access to food and water. All experiments were performed in accordance with the European Communities Council Directive (86/609/EEC) and the United States National Institutes of Health Guide for the care and use of laboratory animals. The protocol “Synaptopathies mesocorticales” n°2015121715284829-V4 n°#3279 was approved by Comité d’Ethique de Marseille. All behavioral and electrophysiological experiments were performed on pubescent and adult rats from both sexes. Male and female rats do not reach puberty at the same time (Schneider, 2013), thus experiments in pubescent animals were performed in male rats between 47 and 51 and female rats between 34 and 37 days of age. Male and female rats were considered adult at PND 90–120. All animals were experimentally naïve and used only once. The number of animals per group is indicated in the corresponding figure legends.

### Drugs

The mixed cannabinoid agonist WIN55,212–2 (WIN; 2 mg/kg) was dissolved in 10% polyethylene glycol/10% Tween80/saline and injected subcutaneously (s.c.) 24 h before the behavioral and electrophysiological essays. Control animals (Sham group) received vehicle. Solutions were freshly prepared on the day of the experiment and were administered in a volume of 2 mL/kg for rats weighing <150 g and 1 mL/kg for adult rats. WIN is a cannabimimetic with a higher affinity for CBRs than THC (Lawston et al., 2000). In rodents, WIN mimics most of the effects elicited by marijuana (Richardson et al., 2002; Viveros et al., 2005). It is estimated that the average content of THC in a joint is 3 mg/kg (Zamberletti et al., 2012). However, as the degree of CB1/CB2 activation after WIN administration at this same dose would be much greater compared to THC, we decided to use a slightly smaller dose. The 2 mg/kg dose chosen for single exposure is within the 1.2–3 mg/kg range that reliably causes behavioral and neuronal effects when given chronically (Tagliaferro et al., 2006; Wegener and Koch, 2009).

### Behavioral Paradigms

Experiments were performed 24 h after WIN or vehicle administration in a sound attenuated chamber under dim light conditions (15–25 lux). Animals were handled two consecutive days before starting the behavioral tests and adapted to the room laboratory conditions 1 h before the tests. They were tested in a 45 × 45 cm Plexiglass arena with ±2 cm of wood shavings covering the floor. Drug treatments were counterbalanced by cage (mates were allocated to different treatment groups). Behavioral procedures were performed between 10:00 am and 3:00 pm. All sessions were recorded using a video camera using the Ethovision XT 13.0 video tracking software (Noldus, Netherlands) and analyzed by a trained observer who was unaware of treatment condition.

### Social Behavior in Pubescent and Adult Rats

The social behavior test was performed as previously published (Manduca et al., 2015). The animals of each pair were equally treated (WIN or vehicle), did not differ more than 10 g in body weight and were sex and age mates but not cage mates. Pubescent or adult rats of both sexes were individually habituated to the test cage daily for either 10 (pubescent) or 5 min (adult) 2 days prior to testing. At the end of the second day of habituation (24 h before the test), the rats received the treatment. To enhance their social motivation and thus facilitate the expression of social behaviors, pubescent and adult animals were socially isolated before testing for 3.5 and 24 h, respectively (Niesink and Van Ree, 1989). The test consisted of placing two equally treated rats into the test cage for either 15 min (pubescent) or 10 min (adult).

In pubescent rats, we scored: 1/Social behavior related to play: pouncing (one animal is soliciting the other to play by attempting to nose or rub the nape of its neck) and pinning (one animal lying with its dorsal surface on the floor with the other animal standing over it). This is the most characteristic posture in social play in rats; it occurs when one animal is solicited to play by its test partner and rotates to its dorsal surface (Panksepp and Beatty, 1980; Trezza et al., 2010) and 2/Social behavior unrelated to play (assessed as a measure of general social interest): sniffing (when the rat sniff, licking, or grooms any part of the body of the test partner).

In adult rats we scored: 1/Play-related behaviors: pouncing, pinning and boxing and 2/Social behaviors unrelated to play: sniffing, social grooming (the rat licks and chews the fur of the conspecific, while placing its forepaws on the back or the neck of the other rat), following/chasing (walking or running in the direction of the partner which stays where it is or moves away), crawling under/over (one animal crawls underneath or over the partner’s body, crossing it transversely from one side to the other), kicking (the rat kicks backwards at the conspecific with one or both hind paws).

The parameters were analyzed grouped and considered as *total social exploration*, calculated as the sum of social behaviors. Aggressive behavior was also scored but not considered in the calculation of *total social exploration*.

### Open Field

The test was performed as previously described (Manduca et al., 2017). Each animal was transferred to the center of the arena and allowed to freely explore for 10 min. The floor was cleaned between each trial to avoid olfactory clues. Numbers of rearing and grooming were manually scored. A video tracking system (Ethovision XT, Noldus Information Technology) recorded the total distance traveled and time spent in the central zone (21 × 21 cm) of the apparatus.

### Novel Object Recognition Test

The test comprised two phases: training (acquisition trial) and test. Each session lasted 5 min. During the acquisition trial, the rat was placed into the arena containing two identical sample objects (A1 and A2) placed near the two corners at either end of one side of the arena (8 cm from each adjacent wall). Thirty minutes later, the rat returned to the apparatus containing two objects, one of them was a copy to the object used in the acquisition trial (A3), and the other one was novel (B). The objects in the test were placed in the same positions as during the acquisition trial. The positions of the objects in the test and the objects used as novel or familiar were counterbalanced between the animals. Exploration was scored when the animal was observed sniffing or touching the object with the nose and/or forepaws. Sitting on objects was not considered to indicate exploratory behavior. The apparatus and the objects were cleaned thoroughly with 50% ethanol between trials to ensure the absence of olfactory cues. The recognition index was calculated as follow: time spent by each animal exploring the novel object divided by the total time spent exploring both objects. Recognition index higher than 0.5 indicates preferable object recognition memory. Number of rearing and grooming were registered during the acquisition trial.

### Slice Preparation

Twenty-four hours after WIN or vehicle administration, rats were anesthetized with isoflurane and decapitated according to institutional regulations. The brain was sliced (300 μm) in the coronal plane with a vibratome (Integraslice, Campden Instruments, Loughborough, UK) in a sucrose-based solution at 4°C (values in mM: 87 NaCl, 75 sucrose, 25 glucose, 5 KCl, 21 MgCl_2_, 0.5 CaCl_2_, and 1.25 NaH_2_PO_4_). Slices were allowed to recover for 60 min at ±32°C in a low calcium artificial cerebrospinal fluid (aCSF; in mM: 126 NaCl, 2.5 KCl, 2.4 MgCl_2_, 1.2 CaCl_2_, 18 NaHCO_3_, 1.2 NaH_2_PO_4_, and 11 glucose, equilibrated with 95% O_2_/5% CO_2_. Slices were maintained at room temperature until recording.

### Electrophysiology

Whole-cell patch-clamp and extra-cellular field recordings were made from layer 5 pyramidal cells of the prelimbic cortex (mPFC; Kasanetz et al., 2013; Martin et al., 2016). For recording, slices were superfused (1.5–2 mL/min) with aCSF containing picrotoxin (100 μM) to block GABA_A_ receptors. All experiments were performed at 32 ± 2°C. To evoke synaptic currents, 100–200 μs stimuli were delivered at 0.1 Hz through an aCSF-filled glass electrode positioned dorsal to the recording electrode in layer 5. Patch-clamp recordings were performed with a potassium gluconate based intracellular solution (values mM: 143 potassium gluconate, 3 NaCl, 1 MgCl_2_, 0.3 CaCl_2_, 1 EGTA, 0.2 cAMP, 0.3 NaGTP, 2 NaATP, 10 HEPES, pH 7.25, osmolarity 290–300 mol/L). Patch pipettes had a resistance between 3 and 5 MΩ. Cells were clamped at −70 mV (without junction potential correction). During recordings holding currents, series and input resistances and the membrane time constant (τ) were monitored. If the series resistance exceeded 25 MΩ or varied by >20% during the experiment the recording was rejected.

Current-voltage (*I–V*) curves were made by a series of hyperpolarizing to depolarizing current steps immediately after breaking into the cell. Membrane resistance was estimated from the *I–V* curve around resting membrane potential (Thomazeau et al., 2014).

For extracellular field experiments, the recording pipette was filled with aCSF. The glutamatergic nature of the field excitatory postsynaptic potential (fEPSP) was systematically confirmed at the end of the experiments using the ionotropic glutamate receptor antagonist 6-cyano-7-nitroquinoxaline-2, 3-dione (CNQX, 20 μM), that specifically blocked the synaptic component without altering the non-synaptic component (data not shown). Example EPSPs and fEPSPs are single sweeps from the indicated time points, for clarity the stimulation artifact was removed from the fEPSP.

### Data Analysis

The magnitude of plasticity was calculated 35–40 min after and compared to the average of baseline response. fEPSP were analyzed with Clampfit 10.6.2.2 (Molecular Devices, LLC). Statistical analysis of data was performed with Prism 6 (GraphPad Software) using tests indicated in the main text after outlier subtraction (ROUT’s test). The number of outliers is indicated in the main text. Statistical tests were chosen based on normality distribution according to D’Agostino-Pearson normality test. Graph values are given as mean ± SEM and table values are given as median and interquartiles ranges. Statistical significance was set at *p* < 0.05 (two-tailed).

## Results

ROUT’s test analysis indicates outliers’ subjects present in some experimental groups as follows. Social behavior test (adults): one subject removed from female WIN group for number of pouncing, number of pinning and number of total social interactions; open field test (adults): one subject removed from Sham Male group for total distance traveled; novel object recognition test (adults): one subject removed from Sham female group for index discrimination; patch-clamp experiments (pubescents): three cells from two different rats removed from Sham female group, one cell removed from WIN female group and two cells from one rat removed from Sham male group for resting potential. Patch-clamp experiments (adults): one cell removed from Sham female group and one cell removed from Sham male group for resting potential. All these cells were then considered as outliers for other parameters such as IV-curve, rheobase and number of spikes.

All the experiments were performed using the mixed synthetic CB1/CB2 receptor agonist WIN55,212-2 that mimics most of the effects elicited by marijuana or THC in rodents (Richardson et al., 2002; Viveros et al., 2005). The 2 mg/kg dose chosen for single exposure is within the 1.2–3 mg/kg range that reliably causes behavioral and neuronal effects when given chronically (Tagliaferro et al., 2006; Wegener and Koch, 2009). The subcutaneous route of administration was chosen to minimize stress.

### Single Exposure to WIN Alters Social Behavior in a Sex- and Age-Dependent Manner

We compared distinct behavioral elements related to the social repertoire of rodents in male and female rats at different ages (puberty and adulthood) previously exposed to a single dose (2 mg/kg) of the synthetic cannabinoid agonist WIN55,212-2 (WIN). In contrast with previous studies where animals were tested shortly after WIN administration, i.e., 30 min after 0.1–1 mg/kg (Trezza and Vanderschuren, 2008a), 0.3 mg/kg (Trezza and Vanderschuren, 2008b) and 1.2 mg/kg (Schneider et al., 2008), the behavioral and synaptic tests were performed 24 h after WIN administration.

**Figure 1:**
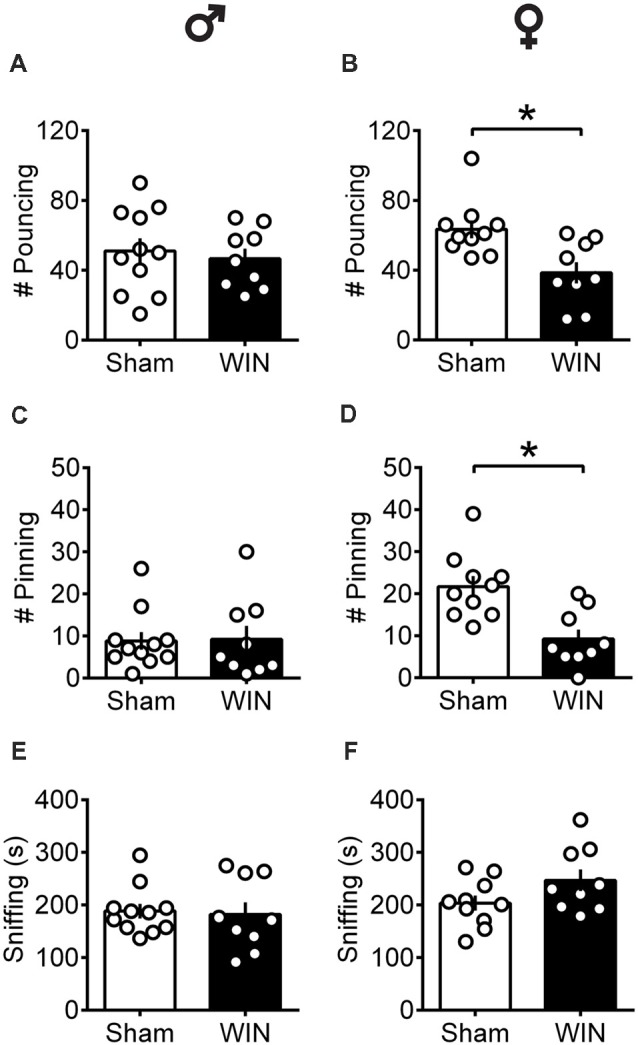
Sex-specific alteration of play behavior in pubescent rats 24 h after a single *in vivo* cannabinoid exposure. Twenty-four hours following a single exposure to WIN55,212-2 (WIN, 2 mg/kg, s.c.), pouncing was normal in male pubescent rats **(A)** in contrast to female littermates **(B)** whom displayed a marked reduction in the number of pouncing compared to Sham animals. Twenty-four hours following WIN exposure, pinning was similar to that of Sham animals in males **(C)** but was largely reduced in female littermates **(D)**. WIN-exposed rats of both sexes (**E**, Male; **F**, Female) spent similar time sniffing the congener compared to their respective Sham groups. Data represent mean ± SEM. Scatter dot plot represents a pair of animals. *p < 0.05, Mann-Whitney *U*-test. ♂ Males (Sham *n* = 11 pairs; WIN *n* = 9 pairs); ♀ Females (Sham *n* = 10 pairs; WIN *n* = 9 pairs).

At puberty, male rats exhibited normal social play behavior 24 h after a SCE: the number of pouncing (Figure 1A: *U* = 44, *p* = 0.696, Mann-Whitney *U*-test) and pinning (Figure 1C: *U* = 42, *p* = 0.588, Mann-Whitney *U*-test) behaviors were unaltered. Accordingly, the total time spent exploring the partner during the test was unaffected (Figure 1E: *U* = 42, *p* = 0.602, Mann-Whitney *U*-test). In contrast, pubescent female rats showed significant reductions on parameters related to play behavior evidenced by a marked reduction in the number of play solicitations, i.e., pouncing (Figure 1B: *U* = 13.5, *p* = 0.008, Mann-Whitney *U*-test) and play responses, i.e., pinning (Figure 1D: *U* = 9, *p* = 0.001, Mann-Whitney *U*-test) observed 24 h after WIN administration. On the other hand, the total time spent exploring the social partner was comparable to that of the Sham group (Figure 1F: *U* = 27, *p* = 0.156, Mann-Whitney *U*-test), indicating a specific impairment on social play behavior in this group. When comparing the male and female Sham groups, a significant difference was found in the number of pinning behaviors (*U* = 11, *p* = 0.001, Mann-Whitney test, data not shown). Females presented higher levels of pinning than males (21.70 ± 2.47 and 8.81 ± 2.10, respectively) which may be attributed to either the sex difference *per se* or the age difference at which puberty appears in both sexes.

**Figure 2:**
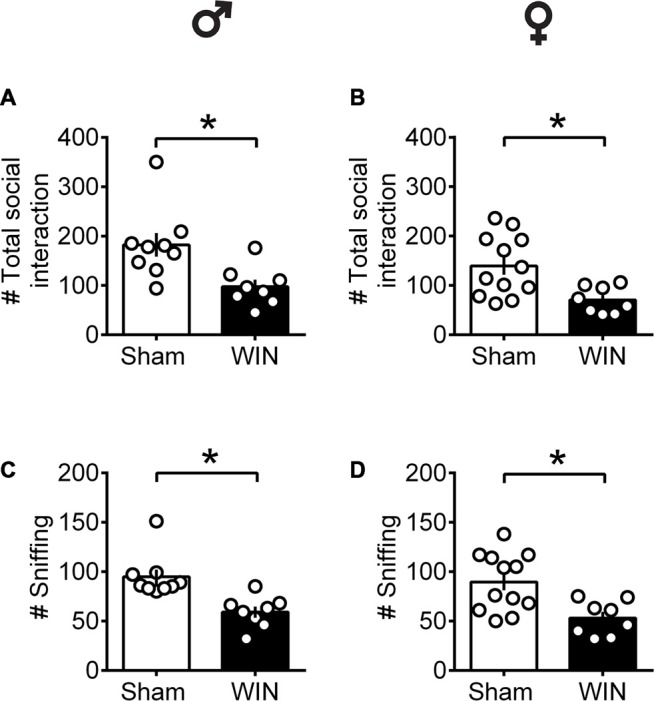
Social interactions are diminished in adult rats of both sex 24 h after a single *in vivo* cannabinoid exposure. Adult male **(A)** and female **(B)** rats had fewer social contacts with their congeners 24 h following a single exposure toWIN55,212-2 (2 mg/kg, s.c.). Similarly, sniffing was reduced in both adult male **(C)** and female **(D)** rats 24 h following a single exposure WIN, compared to control animals. Data represent mean ± SEM. Scatter dot plot represents a pair of animals. *p < 0.05, Mann-Whitney *U*-test. ♂ Males (Sham *n* = 8 pairs; WIN *n* = 8 pairs); ♀ Females (Sham *n* = 12 pairs; WIN *n* = 8 pairs).

In contrast to pubescent rats, both male and female adult rats showed reduced social interest 24 h after SCE. Adult male rats administered WIN presented reduced general social exploration (Figure 2A: *U* = 7, *p* = 0.003, Mann-Whitney *U*-test) as well as reduced sniffing exploration (Figure 2C: *U* = 3.5, *p* < 0.001, Mann-Whitney *U*-test) compared to the Sham group. Similarly, adult cannabinoid-exposed females had less social contact (Figure 2B: *U* = 14.5, *p* = 0.007, Mann-Whitney *U*-test) and sniffing events (Figure 2D: *U* = 15.5, *p* = 0.010, Mann-Whitney *U*-test) with congeners. In addition, SCE did not elicit aggressive behavior in any of the tested groups (data not shown).

Together, these data show that during puberty, SCE is sufficient to alter social behavior in a sex-specific manner: play behavior was specifically reduced in females while males were spared. In adults, SCE caused a general impairment in sociability, exhibited by a reduced number of events related to the total social contacts and sniffing in both male and female rats.

**Figure 3:**
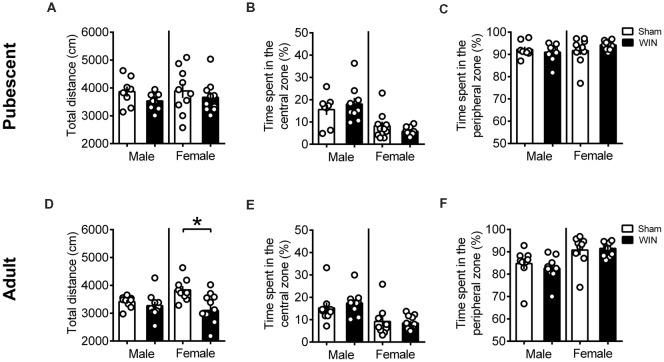
Sex-specific effects in locomotion 24 h after single *in vivo* cannabinoid exposure. Single administration of WIN55,212-2 (WIN, 2 mg/kg, s.c.) 24 h before did not alter the distance traveled **(A)** nor the time spent in the central **(B)** or peripheral zone **(C)** of the apparatus in pubescent rats of both sexes during the open field test. At adulthood, WIN-exposed females had a reduction in the locomotion, while males were not affected **(D)**. Time spent in the central **(E)** or peripheral zone **(F)** remain unchanged in adult rats of both sexes. Data represent mean ± SEM. Scatter dot plot represents one animal. *p < 0.05, Mann-Whitney *U*-test. Pubescent Males: Sham *n* = 8 and WIN *n* = 9; Pubescent Females: Sham = 10 and WIN = 10; Adult Males: Sham = 9 and WIN = 10; Adult Females: Sham *n* = 9 and WIN *n* = 11.

Additional groups of pubescent and adult rats, from either sex, were evaluated in the open field test 24 h after WIN administration. As shown in Figure 3, previous WIN administration did not alter pubescent rats’ behavior in the open field test, since there was no change in the total distance traveled in either male or female groups treated with win (Figure 3A, male: *U* = 20, *p* = 0.138; female: *U* = 39, *p* = 0.435; Mann-Whitney *U*-test). Interestingly, parameters that would suggest alterations on anxiety levels in WIN-treated pubescent rats such as the time spent in the central part of the arena (Figure 3B, male: *U* = 34, *p* = 0.888; female: *U* = 39, *p* = 0.435; Mann Whitney *U*-test) or in the time spent in the peripheral zone (Figure 3C, Male: *U* = 34, *p* = 0.888; female: *U* = 39, *p* = 0.436) remained unchanged 24 h after WIN administration. Furthermore, the number of rearing (male: *U* = 29.5, *p* = 0.138; female: *U* = 43, *p* = 0.615; Mann-Whitney *U*-test) and grooming behaviors (male Sham: 1.62 ± 0.49, male WIN: 2.77 ± 0.49; female Sham: 4.10 ± 1.49, female WIN: 2.60 ± 0.49; mean ± SEM. *U* = 28.5, *p* = 0.487; female: *U* = 45, *p* = 0.723; Mann-Whitney *U*-test; data not shown) was similar between Sham and WIN treated groups of both sexes.

Locomotion was reduced in the adult female group 24 h after WIN administration (Figure 3D, *U* = 24, *p* = 0.029; Mann-Whitney *U*-test), but no change in the time spent in the central part of the apparatus (Figure 3E, *U* = 44, *p* = 0.710; Mann Whitney *U*-test) or in the time spent in the peripheral zone (Figure 3F, *U* = 44, *p* = 0.710; Mann Whitney *U*-test) was observed. Rearing (*U* = 33.5, *p* = 0.136; Mann-Whitney *U*-test) and grooming (*U* = 32, *p* = 0.101; Mann Whitney *U*-test) were not affected by previous WIN exposure in this group (data not shown). Adult male rats exhibited no alteration in the total distance traveled (Figure 3D, *U* = 29, *p* = 0.211; Mann Whitney *U*-test) nor in the time spent in the central (Figure 3E, *U* = 26, *p* = 0.133; Mann Whitney *U*-test) or peripheral zones of the apparatus (Figure 3F, *U* = 26, *p* = 0.133; Mann Whitney *U*-test). As observed in adult females, rearing behavior (*U* = 33.5, *p* = 0.136; Mann-Whitney *U*-test) and grooming (*U* = 25, *p* = 0.298; Mann Whitney *U*-test) were not affected in adult males previously exposed to WIN (data not shown). Taken together, these data do not suggest a major contribution of WIN-induced anxiety to the reduction in sociability observed in adult rats.

### Intact Memory Recognition in Pubescent and Adult Rats of Both Sexes After Single Cannabinoid Exposure

**Figure 4:**
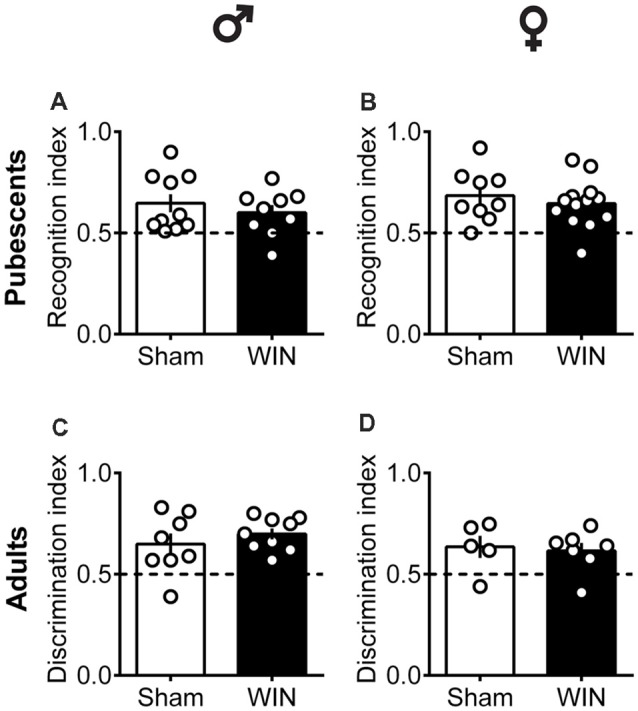
Intact memory discrimination in the novel object recognition test 24 h after single *in vivo* cannabinoid exposure in both pubescent and adult male and female rats. Animals were tested 24 h after a single exposure to WIN55,212-2 (WIN, 2 mg/kg, s.c.). Recognition index between the novel and familiar objects were similar in male **(A)** and female **(B)** WIN-treated pubescent rats compared to their respective Sham groups. Similarly, no differences were observed in recognition index in male **(C)** and female **(D)** adult rats treated with WIN. Data represent mean ± SEM. Scatter dot plot represents one animal. Mann-Whitney *U*-test. ♂ Males (Pubescents: Sham *n* = 10 and WIN *n* = 9; Adults: Sham *n* = 8 and WIN *n* = 9); ♀ Females (Pubescents: Sham *n* = 9 and WIN *n* = 13; Adults: Sham = 5 and WIN = 7).

In humans (Walsh et al., 2017) and rodents (Wegener et al., 2008; Han et al., 2012; Galanopoulos et al., 2014), cannabinoids rapidly impair recent memory. Social behavior requires emotional control and cognitive abilities (Trezza et al., 2014). Thus, we used the Novel Object Recognition test to evaluate the consequences of SCE on rats of our sex and age groups. Twenty four hours after SCE, pubescent male (Figure 4A: *U* = 17, *p* = 0.999, Mann-Whitney *U*-test) and female (Figure 4B: *U* = 52, *p* = 0.682, Mann-Whitney *U*-test) rats presented normal short-term memory. Furthermore, recognition indexes were similar in both adult male and female Sham- and WIN-treated rats (Figure 4C: male, *U* = 29.5, *p* = 0.557; Figure 4D: female, *U* = 15, *p* = 0.755; Mann-Whitney *U*-test). The total time spent exploring the objects during the acquisition trial was not altered in any of the tested groups (Pubescent Males: Sham vs. WIN, *U* = 31, *p* = 0.277; Pubescent Females: Sham vs. WIN, *U* = 42, *p* = 0.292; Adult Males: Sham vs. WIN, *U* = 31, *p* = 0.673; Adult Females: Sham vs. WIN, *U* = 5, *p* = 0.082; Mann-Whitney *U*-test; data not shown). Similarly, none of the parameters linked to exploration and emotionality such as rearing and grooming were altered after WIN administration during the acquisition trial of the test (Table 1).

**Table 1:** Statistical report for rearing and grooming events in pubescent and adult rats from both sexes tested 24 h after a single *in vivo* exposure to WIN55,212-2 (2 mg/kg, s.c.).

		Sham			WIN				
		Median	Quartiles	*n*	Median	Quartiles	*n*	*p*	*U*
Rearing	♂ Pubescent	37.50	32.75/46.00	10	38.00	31.00/43.50	9	0.826	42
	♂ Adult	35.50	25.25/44.00	8	36.00	16.50/40.00	9	0.524	29
	♀ Pubescent	35.00	25.00/40.50	9	37.00	30.50/45.00	13	0.502	48
	♀ Adult	22.00	19.50/30.50	5	28.00	23.00/44.00	7	0.162	8.5
Grooming	♂ Pubescent	0.50	0/2.25	10	2.00	1.00/3.00	9	0.129	26.5
	♂ Adult	1.00	0.25/1.75	8	1	1.00/3.00	9	0.395	26.5
	♀ Pubescent	1.00	0/2.50	9	1	1.00/5.00	13	0.732	53
	♀ Adult	2.00	0.50/4.50	5	3.00	0/5.00	7	0.977	17

*The number of rearing and grooming was counted during the acquisition trial in the novel object recognition test. Quartiles, 25 and 75% percentiles; n, number of animals; Mann-Whitney *U*-test; ♂ males; ♀ females*.

### Single *in vivo* Cannabinoid Exposure Leads to Sex-Specific Ablation of Prefrontal eCB Plasticity

The central position of the PFC and eCB system in the regulation of social behavior and the important role of synaptic plasticity in this structure in mediating experience-dependent adaptations are well-documented (for review see Araque et al., 2017). At the synaptic level, activity-dependent plasticity in the PFC—including eCB-mediated LTD and NMDAR-mediated LTP—is a common target in animal models of neuropsychiatric diseases (Scheyer et al., 2017). We compared the LTD mediated by the eCB system (eCB-LTD) in the PFC between Sham- and WIN-treated rats of both sexes at different ages, specifically pubescence and adulthood.

**Figure 5:**
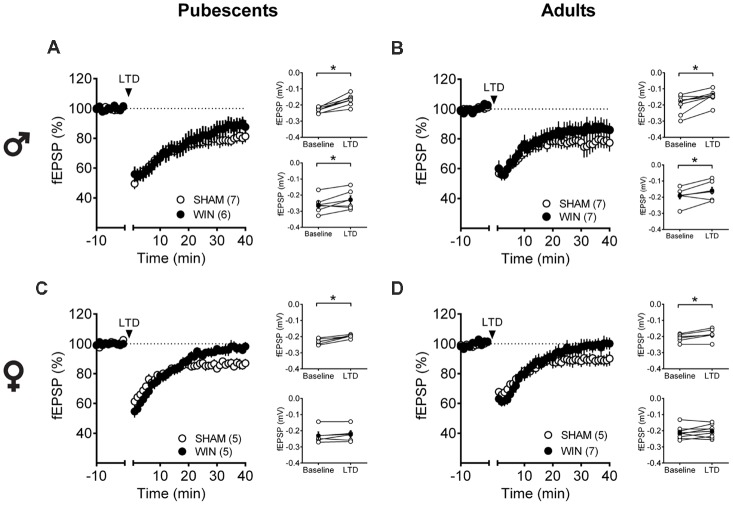
Sex-specific effects of a single *in vivo* cannabinoid exposure on prefrontal cortex (PFC) endogenous cannabinoid (eCB)-long-term depression (LTD). Rats were exposed to WIN55,212-2 (WIN, 2 mg/kg, s.c.) 24 h before. Average time-courses of mean field excitatory postsynaptic potentials (fEPSPs) showing that low-frequency stimulation (indicated by arrow) induced LTD at mPFC synapses in both Sham- (white circles, *n* = 7) and WIN- (black circles, *n* = 6) exposed pubescent males **(A)**. Similarly, LTD was identical in Sham- (white circles, *n* = 7) and WIN- (black circles, *n* = 7) exposed adult males **(B)**. In contrast, LTD was ablated in mPFC slices obtained from both pubescent (**C**, Sham, white circles, *n* = 5; WIN, black circles, *n* = 5) and adult (**D**, Sham, white circles, *n* = 5; WIN, black circles, *n* = 7) females 24 h after a single exposure to WIN. Adjacent to the time-course figures individual experiments (white circles) and group average (Sham, gray circles; WIN, black circles) before (baseline) and after (35–40 min) LTD induction are showed. LTD is present in WIN-treated male rats at both ages: pubescent (**A**, on the right) and adulthood (**B**, on the right). In contrast, LTD was absent in both pubescent (**C**, on the right) and adult (**D**, on the right) females previously treated with WIN. Error bars indicate SEM, *n* = individual rats, *p < 0.05, Paired *t*-test. ♂ Males; ♀ Females.

Low-frequency stimulation of layer 5 PFC synapses induced comparable LTD in both control and cannabinoid-exposed pubescent male rats (Figure 5A: Sham: *t*_(6)_ = 5.596, *p* = 0.001; WIN: *t*_(4)_ = 3.190, *p* = 0.033; Paired *t*-test). Similar results were observed in adult males with or without prior *in vivo* cannabinoid exposure (Figure 5B: Sham, *t*_(6)_ = 3.116, *p* = 0.020; WIN, *t*_(6)_ = 2.787, *p* = 0.031; Paired *t*-test). In the male rat PFC, eCB-LTD is not affected 24 h after *in vivo* cannabinoid administration. Strikingly, eCB-LTD was ablated in PFC slices obtained from female rats in both age groups. Figure 5C shows the lack of LTD in PFC slices from cannabinoid-treated pubescent (Sham, *t*_(4)_ = 5.021, *p* = 0.007; WIN, *t*_(4)_ = 1.129, *p* = 0.322; paired *t*-test) and adult female rats (Figure 5D: Sham, *t*_(4)_ = 2.979, *p* = 0.040; WIN, *t*_(7)_ = 1.003, *p* = 0.349; paired *t*-test).

### Age- and Sex-Dependent Ablation of LTP After *in vivo* Single Exposure to Cannabinoid

**Figure 6:**
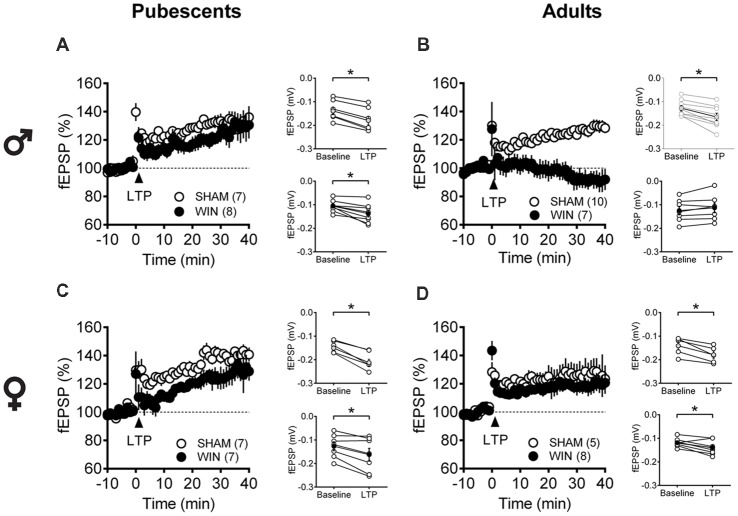
Age- and sex-dependent ablation of long-term potentiation (LTP) in the rat PFC 24 h after *in vivo* cannabinoid exposure. Rats were exposed to WIN55,212-2 (WIN, 2 mg/kg, s.c.) 24 h before. Average time-courses of mean fEPSPs showing that theta-burst stimulation (indicated arrow) induced a LTP at mPFC synapses in both Sham- (white circles, *n* = 7) and WIN- (black circles, *n* = 8) exposed pubescent males **(A)** but not WIN-treated adults (**B**, Sham: white circles, *n* = 10; WIN: black circles, *n* = 7). In contrast, LTP was present in mPFC slices obtained from both pubescent (**C**, Sham: white circles, *n* = 7; WIN: black circles, *n* = 7) and adult (**D**, Sham: white circles, *n* = 5; WIN: black circles, *n* = 8) WIN-treated females. Adjacent to the time-course figures are showed individual experiments (white circles) and group average (Sham, gray circles; WIN, black circles) before (baseline) and after (35–40 min) LTP induction showing that, in males, LTP is present in pubescent (**A**, on the right) but not in adults (**B**, on the right). In contrast, LTP was present in both pubescent (**C**, on the right) and adult (**D**, on the right) females previously treated with WIN. Error bars indicate SEM, *n* = individual rats, *p < 0.05, Paired *t*-test. ♂ Males; ♀ Females.

Considering that the extensive repertoire of synaptic plasticity expressed by medial PFC synapses is sensitive to various regimens of exposure to drugs of abuse (Kasanetz et al., 2013; van Huijstee and Mansvelder, 2015; Lovelace et al., 2015; Renard et al., 2016a; Cannady et al., 2017), we assessed a second type of plasticity in the PFC which is frequently related to endophenotypes of neuropsychiatric disorders (Thomazeau et al., 2014; Neuhofer et al., 2015; Iafrati et al., 2016; Labouesse et al., 2017; Manduca et al., 2017), the NMDAR-dependent LTP (NMDAR-LTP). NMDAR-LTP was ablated in adult male rats while pubescent males were spared. Figures 5A,B show comparable LTP between Sham and cannabinoid-treated pubescent male rats (Figure 6A: Sham, *t*_(6)_ = 9.676, *p* < 0.001; WIN, *t*_(7)_ = 3.677, *p* = 0.007; Paired *t*-test), but not in adult male rats (Figure 6B: Sham, *t*_(8)_ = 5.560, *p* < 0.001; WIN, *t*_(6)_ = 2.062, *p* = 0.084; Paired *t*-test). In contrast, in both age groups, NMDAR-LTP was comparable in Sham- and cannabinoid-treated female rats: both pubescent (Figure 6C: Sham, *t*_(6)_ = 8.424, *p* < 0.001; WIN, *t*_(6)_ = 3.369, *p* = 0.015; Paired *t*-test) and adult rats (Figure 6D: Sham, *t*_(4)_ = 4.349, *p* = 0.012; WIN, *t*_(7)_ = 3.133, *p* = 0.016; Paired *t*-test) had normal NMDAR-LTP 24 h following *in vivo* cannabinoid exposure.

### Single *in vivo* Exposure to WIN Causes Age- and Sex-Specific Modifications in Intrinsic Pyramidal Neuron Properties

**Figure 7:**
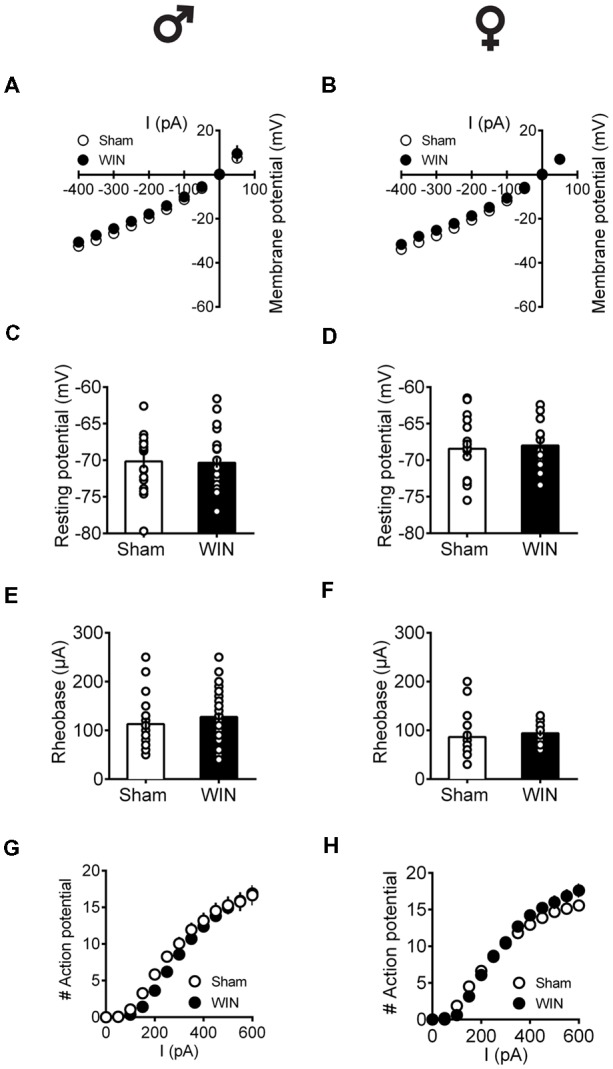
Intrinsic properties of PFC pyramidal neurons are not altered by single *in vivo* exposure to cannabinoid in pubescent rats.Rats received a single administration of WIN55,212-2 (WIN, 2 mg/kg, s.c.) 24 h before. Current–voltage plot from visually identified pyramidal neurons recorded from pubescent rats showing similar cell voltage in response to current steps between Sham and WIN of both male **(A)** and female **(B)** groups. No change was observed in the resting membrane potential 24 h after WIN treatment in both male **(C)** and female **(D)** pubescent rats. Quantification of neuronal spiking properties indicated no change in the rheobase of either males **(E)** or females **(F)** 24 h after single WIN. The number of evoked action potentials in response to increasing depolarizing current steps was similar in Sham and WIN-treated male **(G)** and female **(H)** pubescent rats. Males: Sham, *n* = 15 cells/6 rats; WIN, *n* = 28 cells/10 rats. Females: Sham, *n* = 17 cells/7 rats; WIN, *n* = 13 cells/7 rats. Scatter dot plot represents one cell. Data represent mean ± SEM. ♂ Males; ♀ Females.

Independent of sex, all recorded PFC neurons in pubescent rats showed similar membrane reaction profiles in response to a series of somatic current steps 24 h after SCE (Figure 7A: male, *F*_(interaction 10,440)_ = 1.551, *p* = 0.118; Figure 7B: female, *F*_(interaction 10,270)_ = 0.499, *p* = 0.889; two-way repeated-measures ANOVA). The resting membrane potential (Figure 7C: male, *U* = 230, *p* = 0.627; Figure 7D: female, *U* = 99.5, *p* = 0.854; Mann-Whitney *U*-test), as well as the rheobase (Figure 7E: male, *U* = 194.5, *p* = 0.198; Figure 7F: female, *U* = 68, *p* = 0.115; Mann-Whitney *U*-test), were comparable between Sham- and WIN-treated pubescent rats from both sexes. Also, no changes in excitability were observed since the number of actions potentials in response to somatic currents steps were comparable in both control and WIN-treated pubescent rats of both sexes (Figure 7G: male, *F*_(interaction 12,492)_ = 1.189, *p* = 0.287; Figure 7H: female, *F*_(interaction 12,324)_ = 3.624, *p* < 0.001 and *F*_(treatment 1,27)_ = 0.389, *p* = 0.537; two-way repeated measures ANOVA).

**Figure 8:**
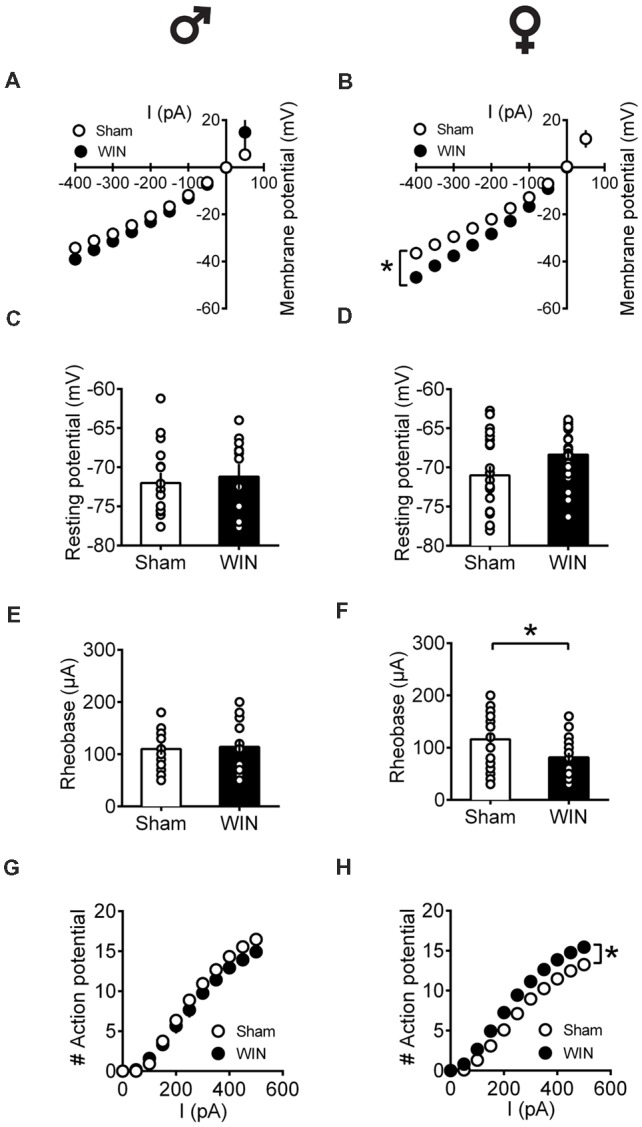
Sex-specific alteration of pyramidal neurons’ intrinsic properties in adult rats 24 h following single *in vivo* cannabinoid administration. Rats received a single administration of WIN55,212-2 (WIN, 2 mg/kg, s.c.) 24 h before. Current–voltage plot from visually identified pyramidal neurons recorded from adult rats showing no difference in cell voltage in response to current steps between Sham and WIN groups of adult male rats **(A)**. In contrast, membrane potentials were altered in adult WIN-treated females compared to control group **(B)**. The resting membrane potentials were similar to that of control in adult males **(C)** and females **(D)** 24 h following single WIN exposure. Quantification of neuronal spiking properties showed no change in the rheobase of males **(E)**, but a marked reduction in the female WIN-treated group **(F)**. The number of evoked action potentials in response to increasing depolarizing current step was similar in Sham and WIN-treated males **(G)**. In contrast, females showed a higher number of action potentials 24 h after WIN treatment **(H)**. Male: Sham, *n* = 16 cells/6 rats; WIN, *n* = 12 cells/5 rats; Female: Sham, *n* = 16 cells/6 rats; WIN, *n* = 20 cells/7 rats. Scatter dot plot represents one cell. Data represent mean ± SEM. *p < 0.05, Mann-Whitney test (B), Bonferroni’s multiple comparisons test (F). ♂ Males; ♀ Females.

In adult rats however, sex-specific modifications of the excitability of pyramidal neurons sampled from females were observed following a single *in vivo* cannabinoid exposure. Intrinsic properties of layer V PFC pyramidal neurons (I/V curve Figure 8A: *F*_(interaction 9,225)_ = 1.907, *p* = 0.052, two-way repeated measures ANOVA), resting membrane potentials (Figure 8C: *U* = 79, *p* = 0.614, Mann-Whitney *U*-test; rheobase Figure 8E: *U* = 79, *p* = 0.614, Mann-Whitney *U*-test) and the number of action potentials in response to increasing depolarizing current (Figure 8G: *F*_(interaction 10,250)_ = 1.417, *p* = 0.173, two-way repeated measures ANOVA) were comparable in control and WIN-treated male rats. In striking contrast, a single *in vivo* cannabinoid exposure increased the excitability of PFC pyramidal neurons of adult females. Thus, we observed an alteration of the membrane reaction profile in response to a series of somatic current steps (Figure 8B: *F*_(interaction 9,369)_ = 3.480, *p* < 0.001 and *F*_(treatment 1,41)_ = 5.576, *p* = 0.023, two-way repeated measures ANOVA) and a marked reduction of the rheobase (Figure 8F: *U* = 137.5, *p* = 0.023, Mann-Whitney *U*-test) accompanying an increased number of action potential in response to increasing depolarizing current (Figure 8H: *F*_(interaction 10,410)_ = 3.038, *p* = 0.001 and *F*_(treatment 1,41)_ = 8.041, *p* = 0.007, two-way repeated measures ANOVA). The resting membrane potentials were similar to that of control female rats (Figure 8D, *U* = 166.5, *p* = 0.124, Mann-Whitney *U*-test). Taken together, these data suggest an overall increase in the excitability of PFC pyramidal neurons in adult females 24 h after SCE.

## Discussion

We found that 24 h after a single *in vivo* exposure to a cannabinoid, the behavioral, neuronal and synaptic consequences differ depending on the sex and age of the rat. The current data indicate a heightened sensitivity of females, especially during pubescence. Specifically, social behavior and eCB-mediated LTD showed strong deficits in exposed pubescent females while age-matched male littermates were spared. During adulthood, although reduced social interactions were observed in both sexes, eCB-mediated synaptic plasticity was ablated specifically in females and NMDAR-dependent LTP specifically in males.

Stimulation of CB1R acutely modulates social play in adolescent rats (Trezza and Vanderschuren, 2008a). We showed that a single exposure to the synthetic cannabinoid WIN (2 mg/kg), at a dose reported to acutely decrease social interactions in male rats (Schneider et al., 2008; Trezza and Vanderschuren, 2008a,b) has sex-specific effects as long as 24 h after *in vivo* exposure. In the pubescent group, cannabinoid-treated females exhibited less social play behavior but normal social investigation and locomotion while the sociability of pubescent males exposed to WIN was indistinguishable from that of sham rats.

Sham pubescent females presented higher levels of play than sham pubescent males. This observation can be explained by the difference in the age range at which males and females were herein tested: the objective was verifying the effect of acute cannabinoid exposure during pubescence. Thus, as females reach puberty before males (Schneider, 2008), we used females at an earlier PND of development than males. The frequency and intensity of play behaviors peak between PNDs 28–40 regardless of sex (Panksepp, 1981) and decline thereafter when rats reach sexual maturity. Because the latter differs between sexes, it is expected that males and females display different levels of play during puberty. Thus, considering that in our conditions the play behavior of pubescent females was reduced 24 h after SCE to the same levels of those observed in control males, it is tempting to hypothesize that SCE induces a “masculinization” of female social play behavior. Interestingly, a recent study showed that the activation of both CB1 and CB2 receptors (as that observed following exposure to WIN) is implicated in the masculinization of play behavior of pre-pubertal female rats (Argue et al., 2017), reinforcing the idea of sex-dependent modulation of social behaviors that arises early in life. Potential mechanisms include dendrites and spines’ morphological—and presumably functional—alterations (Argue et al., 2017) and/or altered levels of circulating eCB (Craft et al., 2013). Future studies of anhedonia in the current particular experimental condition will provide insights into the rewarding component of social interactions and, for example, rule out putative depressive effect of SCE. Taken together these data confirm and extend those of Craft et al. (2013) who showed that females are more affected by exogenous cannabinoids during pubescence than males.

Gonadal steroids hormones seem to be involved in the sexual differentiation of cannabinoid sensitivity. Importantly, rat hormonal status (i.e., estrous cycle phase) has been reported to significantly influence sex differences for cannabinoid effects (revised from Cooper and Craft, 2018). Indeed, sex differences are not entirely consistent across studies regarding differences in CB1R mRNA or binding affinity and eCB content (Reich et al., 2009; Riebe et al., 2010; Castelli et al., 2014; Weed et al., 2016), supporting the important role of hormonal status in these differences. As cannabinoid dose, administration route, post-administration intervals and rat strains are not consistent among studies, methodological details may help explain this divergence. Comparing the effect of a single administration of WIN during the different phases of the female cycle may help in better understanding the subtle effects reported in the current study.

It is well described that social interactions during adolescence are crucial for the development of social competence at adulthood (Douglas et al., 2004; Vanderschuren and Trezza, 2014) and that modification of rat adolescent social activity alters neurobehavioral parameters related to pain processing, anxiety, depression and substance abuse (reviewed from Burke et al., 2017). Among the several forms of sociability in rodents, play behavior is thought to be of principal importance for social development. When developing rats are deprived from play with sex- and age-matched conspecifics, abnormal patterns of social, sexual and aggressive behaviors are observed once they reach adulthood (Vanderschuren and Trezza, 2014). Future experiments will aid in determining if the deficits caused by SCE are long lasting and whether they can be reversed with pharmacological intervention.

As rats reach sexual maturation, neural alterations of the “social brain” are differently regulated according to age and sex. Thus, the social behavior repertoire differs between pubescent and adult rats (Panksepp and Beatty, 1980; Pellis and Pellis, 2007; Graham and Burghardt, 2010). Accordingly, we showed that acute WIN administration in adult rats triggered different consequences on behavior. In contrast to the pubescent groups, a unique exposure to WIN perturbed social behavior in both sexes at adulthood. There is strong evidence of an anxiolytic/anxiogenic component which influences adult social behaviors. Namely, a decrease in social interactions represents an anxiogenic response while increased social interaction followed by unchanged motor activity indicates an anxiolytic effect (File and Seth, 2003). Parameters that would suggest alterations on anxiety levels in our study such as time spent in the central part of the arena remained unchanged in both males and females treated with WIN. The reduced locomotion observed in adult WIN-treated females could be indicative of a slight sedative effect of WIN, which could in turn be implicated in the reduced sociability herein observed.

Although sex differences on cannabinoids’ effects on cognition have been reported (Rubino et al., 2008; Trezza and Vanderschuren, 2009; Rubino and Parolaro, 2015; Silva et al., 2016; Wiley et al., 2017), some studies demonstrated no changes in learning ability 24 h after cannabinoid administration (water maze, object location and object recognition tasks, WIN 1.2 mg/kg, i.p.; Abush and Akirav, 2012). In the present work, novel object recognition memory was unaffected in either sex, in favor of the idea that the deficits are not generalized but rather selective to the social behavior.

We observed impairments on synaptic plasticity in a sex- and age-dependent manner. Surprisingly, we showed that 24 h after SCE, pubescent males did not display behavioral or synaptic changes, while adult rats did. On the other hand, female rats of both ages were negatively impacted by previous WIN administration, in agreement with the literature showing that pubescent females are the more vulnerable group (Rubino and Parolaro, 2011, 2016; Craft et al., 2013; Renard et al., 2016a). The eCB signaling machinery is positioned in a way to influence PFC communication and control other brain regions (Domenici et al., 2006; Hill et al., 2007). Chronic cannabinoid exposure significantly impairs synaptic plasticity throughout the brain (Renard et al., 2016b; Araque et al., 2017), while the synaptic plasticity deficits resulting from acute cannabinoid exposure largely depend on the brain area. For example, a single exposure to THC (3 mg/kg; 15–20 h before) ablated eCB-mediated synaptic plasticity in the adult mouse NAc and hippocampus (Mato et al., 2004) but not hippocampal CA1 LTP (10 mg/kg; 24 h before; Hoffman et al., 2007) nor eCB-LTD at VTA GABA synapses (Friend et al., 2017). In rats, an acute single injection of WIN (1.2 mg/kg; 24 h before) impaired LTP in the ventral subiculum-accumbens pathway (Abush and Akirav, 2012) and in the Schaffer collateral-CA1 projection (WIN 0.5 mg/kg; 30 min before; Abush and Akirav, 2010). In a dose-dependent manner, WIN (0.5–2 mg/kg, i.p.) impaired short-term plasticity and LTP at perforant path dentate gyrus synapses in adult rats (Colangeli et al., 2017). It is important to highlight that in the aforementioned studies only male rodents were evaluated. Here, we showed that, male rats of both ages showed normal eCB-LTD, the latter was ablated in female rats 24 h after SCE regardless of the age. Furthermore, only adult females exhibited altered neuronal excitability. These results suggest impaired cannabinoid signaling with possible mechanisms ranging from the reduction of presynaptic mobility of surface CB1R receptors as shown by our group 24 h after WIN *in vitro* (Mikasova et al., 2008), sex-differential CB1R desensitization (Farquhar et al., 2019), modified interactions with adaptor proteins (e.g., GASP and AP-3; for review see Howlett et al., 2010) or CB1R interacting proteins (e.g., SGIP1 and CRIP1, Howlett et al., 2010; Hájková et al., 2016) and altered functions of the enzymes controlling circulating eCB.

Sex differences in the eCB system may be involved in the aforementioned effects. Cortical CB1R expression and function are higher in juvenile male rats (PND 28–35) as compared to adolescent sex-matched subjects (PND 40), and CB1R levels decrease thereafter towards young adult levels (PND 70; Heng et al., 2011). Compared to females, male rats have a higher density of CB1R. However, a higher G-protein activation after CB1R stimulation is observed in adolescent females in several brain areas (Rubino et al., 2008; Burston et al., 2010). Additional molecular mechanisms may help explain the observed sex differences. Sexual differences in the eCB system appear early in development in rodents (Craft et al., 2013). Sexually dimorphic regulation of synaptic plasticity or intrinsic neuronal activity in the amygdala (Fendt et al., 2013; Chen et al., 2014; Bender et al., 2017), hippocampus (Huang and Woolley, 2012; Inoue et al., 2014; Harte-Hargrove et al., 2015; Qi et al., 2016) and PFC (Nakajima et al., 2014; Li et al., 2016) has been described. Female rats exhibit greater concentrations of the metabolic enzymes monoacylglycerol lipase (MAGL) and fatty acid amide hydrolase (FAAH) as early as PND 4 compared to males (Krebs-Kraft et al., 2010). Moreover, CB1R expression reaches its peak earlier in females (PND 30) than in males (PND 40; Romero et al., 1997), whereas at adulthood, CB1R density is lower in the PFC and amygdala of cycling females (Castelli et al., 2014). Finally, it was recently shown that the CB1 agonist CP 55, 940 has widespread effects on the brain lipidome in adolescent female mice (Leishman et al., 2018). Thus, females’ eCB systems appear more sensitive to the deleterious influence of exogenous cannabinoids.

While eCB-LTD in both pubescent and adult males was unaffected by a single WIN exposure, NMDAR-LTP was selectively ablated in adult males. Regarding pubescent rats, both males and females were spared. These findings show that cannabinoid-induced impairments on synaptic plasticity are not generalized in the PFC. Altered CB1R activity induced by SCE may be involved in the altered NMDAR-LTP found in adult males. The eCB system controls NMDAR activity through mechanisms involving signaling pathways and/or direct physical coupling between CB1R and NMDAR NR1 subunits (Rodríguez-Muñoz et al., 2016). Some evidence indicates that NMDAR activity may be differently modulated according to sex. Gonadectomy alters mouse behavioral responses to the NMDAR agonist MK-801 in males but not females; male mice have higher NMDAR density in the NAc, motor and cingulate cortices (van den Buuse et al., 2017) and gonadal hormonal status influences both LTP induction and NMDAR function in male rats (Moradpour et al., 2013). Thus, SCE causes similar behavioral deficits in both male and female rats but triggered different alterations of PFC synapses.

Along with sex-specific responses to cannabinoids, sex differences in drug pharmacokinetics may be involved in the reported behavioral and electrophysiological effects. As reviewed by Rubino and Parolaro (2011), dimorphism in the eCB system and in cannabinoid metabolism may explain the different sensitivity between sexes found in which females seem to be more vulnerable to exogenous cannabinoid exposure. Furthermore, potential age-specific differences in the pharmacokinetics of cannabinoids might also explain why pubescent males respond differently to WIN when compared to adults. Even using the same drug dose and administration route, the subsequent mechanisms involved in drug absorption, distribution (specially in brain tissue), metabolism and elimination may not be equivalent between young and adult rodents. In humans, pharmacokinetics parameters such as absorption, volume of distribution, drug bioavailability and clearance are age-related (Fernandez et al., 2011). While age-dependent differences in cannabinoid metabolism cannot completely be ruled out, there is to the best of our knowledge, no report of such an occurrence in the literature.

The present model of subcutaneous WIN injection was chosen over the more common intraperitoneal route to minimize stress to the animals (Stuart and Robinson, 2015). Unfortunately, the pharmacokinetics of WIN and other cannabimimetics after subcutaneous administration remains poorly described (Fox et al., 2001; Carlier et al., 2018). In particular, the presence and distribution of potential active metabolites is not known. Available data show that the half-life of WIN is limited following intraperitoneal injection (Barna et al., 2009) and it is unlikely that the previous results can be explained by ongoing occupation of CB1R by WIN 24 h after subcutaneous exposure. Further experiments during precise stages of the estrous cycle will be necessary to further establish the relationship between CBR activation and gonadal hormones. Finally, taking into consideration the rewarding component of social interactions (Vanderschuren et al., 2016), investigating the role of WIN in the hedonic valence of social behavior will help elucidate the bases of the phenotype observed here.

Together, our results reveal behavioral and synaptic sex differences in response to a single *in vivo* exposure to a cannabinoid. Further analyses of both electrophysiological function and its molecular underpinnings associated with the heightened sensitivity of females to a single *in vivo* exposure to cannabinoid may reveal long-term consequences of these early life drug-induced alterations.

## Author Contributions

MB, AM, AB, A-LP-A and OM designed the research. MB, AM, AB and OL performed the research. MB analyzed the data. MB, A-LP-A and OM wrote the article. OM and A-LP-A supervised the entire project.

## Conflict of Interest Statement

The authors declare that the research was conducted in the absence of any commercial or financial relationships that could be construed as a potential conflict of interest.
